# Adult Renal Stem/Progenitor Cells Can Modulate T Regulatory Cells and Double Negative T Cells

**DOI:** 10.3390/ijms22010274

**Published:** 2020-12-29

**Authors:** Claudia Curci, Angela Picerno, Nada Chaoul, Alessandra Stasi, Giuseppe De Palma, Rossana Franzin, Paola Pontrelli, Giuseppe Castellano, Giovanni B. Pertosa, Luigi Macchia, Vito Francesco Di Lorenzo, Carlo Sabbà, Anna Gallone, Loreto Gesualdo, Fabio Sallustio

**Affiliations:** 1 Nephrology, Dialysis and Transplantation Unit, DETO, University of Bari “Aldo Moro”, 70124 Bari, Italy; claudia.curci@uniba.it (C.C.); angelapicerno89@gmail.com (A.P.); alessandra.stasi@uniba.it (A.S.); rossana.franzin@uniba.it (R.F.); paola.pontrelli@uniba.it (P.P.); giovannibattista.pertosa@uniba.it (G.B.P.); loreto.gesualdo@uniba.it (L.G.); 2 Department of Basic Medical Sciences, Neuroscience and Sense Organs, University of Bari “Aldo Moro”, 70124 Bari, Italy; anna.gallone@uniba.it; 3 Allergology Unit, DETO, University of Bari “Aldo Moro”, 70124 Bari, Italy; nadach@gmail.com (N.C.); luigi.macchia@uniba.it (L.M.); 4 Institutional BioBank, Experimental Oncology and Biobank Management Unit, IRCCS Istituto Tumori “Giovanni Paolo II”, 70124 Bari, Italy; g.depalma@oncologico.bari.it; 5 Nephrology, Dialysis and Transplantation Unit, Department of Medical and Surgical Science, University of Foggia, 71122 Foggia, Italy; giuseppe.castellano@unifg.it; 6 Urology Unit, IRCCS Istituto Tumori “Giovanni Paolo II”, 70124 Bari, Italy; vitodilorenzo1957@gmail.com; 7 Department of Interdisciplinary Medicine, University of Bari “Aldo Moro”, 70124 Bari, Italy; carlo.sabba@uniba.it

**Keywords:** stem cells, immunomodulation, tregs, DN T cells, renal diseases

## Abstract

Adult Renal Stem/Progenitor Cells (ARPCs) have been recently identified in the human kidney and several studies show their active role in kidney repair processes during acute or chronic injury. However, little is known about their immunomodulatory properties and their capacity to regulate specific T cell subpopulations. We co-cultured ARPCs activated by triggering Toll-Like Receptor 2 (TLR2) with human peripheral blood mononuclear cells for 5 days and 15 days and studied their immunomodulatory capacity on T cell subpopulations. We found that activated-ARPCs were able to decrease T cell proliferation but did not affect CD8^+^ and CD4^+^ T cells. Instead, Tregs and CD3^+^ CD4^-^ CD8^-^ double-negative (DN) T cells decreased after 5 days and increased after 15 days of co-culture. In addition, we found that PAI1, MCP1, GM-CSF, and CXCL1 were significantly expressed by TLR2-activated ARPCs alone and were up-regulated in T cells co-cultured with activated ARPCs. The exogenous cocktail of cytokines was able to reproduce the immunomodulatory effects of the co-culture with activated ARPCs. These data showed that ARPCs can regulate immune response by inducing Tregs and DN T cells cell modulation, which are involved in the balance between immune tolerance and autoimmunity.

## 1. Introduction

Adult Renal stem/progenitor cells (ARPCs) can contribute to the kidney regeneration both directly differentiating and by paracrine secretion. They are able to differentiate toward epithelial-, endothelial-, osteogenic-, and adipogenic-like cells [1,2,3] Some studies showed that ARPCs can regenerate long portions of renal tubules and can regenerate lost podocytes in cortical nephrons [4]. Moreover, these CD133+ renal progenitors are activated by Toll-Like Receptor 2 (TLR2) ligands and can secrete reparative factors able to repair renal tubular cells damaged by chemical agents such as the cisplatin [5]. The TLR2 can function as a sensor of the damage, and its activation can produce different effects such as stem cell proliferation and differentiation. Following appropriate TLR2 stimulation, there is a release of cytokines and inflammatory chemokines such as Complement component C3 and MCP-1, IL-6, and IL-8 ([1,6,7,8].

Renal progenitors can repair both a physical damage, such as a wound in the epithelial tissue, and damage produced by a chemical agent such as cisplatin, a widely used chemotherapeutic agent that can have nephrotoxicity side effects. ARPCs include secreted inhibin-A and decorin following renal tubular cell damage, and these secreted factors are directly involved in the cell regeneration process [1]. ARPCs also have the capacity to prevent the endothelial dysfunction and to protect the endothelial compartment following lipopolysaccharide (LPS) exposure, thereby promoting kidney repair. The antifibrotic effect of ARPCs is exercised by the secretion of CXCL6, SAA4, and BPIFA2 antiseptic molecules [9].

Generally, several kind of stem/progenitor cells can also repair tissues by modulating inflammation and regulating the immune response in reaction to an injury [10,11].

Numerous studies showed that mesenchymal stem cells (MSCs) secrete an abundant quantity of immunoregulatory factors and of growth factors facilitating the tissue repair by using resident tissutal cells [12,13]. 

MSC immunosuppressive capacity is not constitutive but it is prompted by other inflammatory cytokines, such as those in the inflammatory microenvironment [14].

It is commonly believed that the beneficial effect of MSCs is principally a result of immunomodulation, which in turn engages other cells to accelerate tissue repair, and that this function is triggered by inflammation [15]. Accordingly, in response to inflammatory molecules, MSCs produce a large quantity of immunoregulatory factors, cell-mobilization factors, and growth factors and thereby facilitate tissue repair by tissue-resident stem cells [12,15]. The MSCs suppress immunoreactions by a concerted action of Nitric oxide (NO) and some specific chemokines [14]. These include interleukin 6 (IL-6), transforming growth factor-B (TGF-B), prostaglandin E2, HGF, an epidermal growth factor, and a fibroblast growth factor [16,17]. The involvement of NO and inducible nitric oxide synthases (iNOS) in MSCs seems to be necessary to obtain the MSC-mediated immunosuppression [18]. NO also plays a central role in the regulation of homeostasis and functions of different adult/progenitor stem cells [19]. In renal injury, ARPCs differentiation toward a myofibroblast-like phenotype is mediated by oxidative stress and by NOX4, a NADPH oxidase renal isoform overexpressed in the presence of reactive oxygen species (ROS) and NO [20,21] 

However, the immunomodulatory potential of ARPCs is not known. Here we studied the ARPCs’ capacity to regulate specific T cell subpopulations with the aim to understand whether they have immunomodulatory properties on human T cells. 

## 2. Results

### 2.1. ARPCs Decrease PBMC Proliferation through TLR2 Triggering

**Figure 1:**
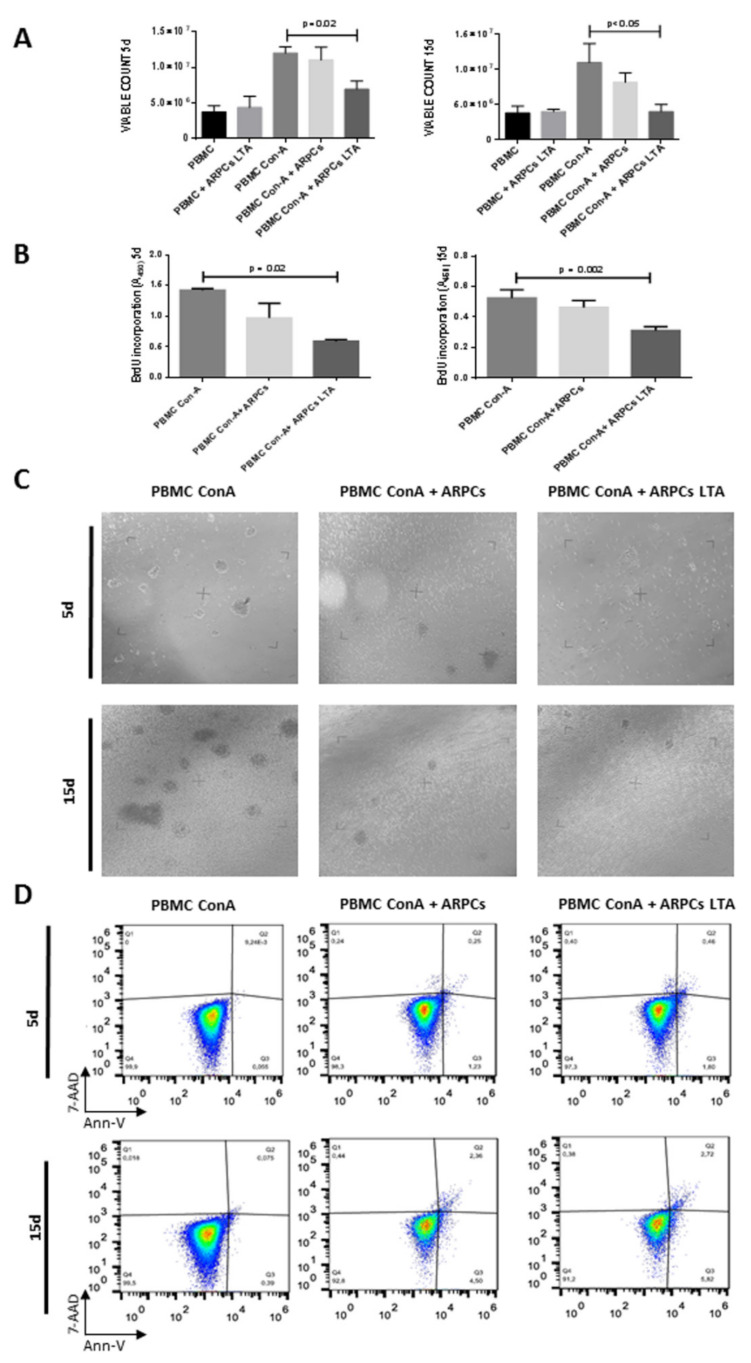
TLR2-activated- Adult Renal Stem/Progenitor Cells (ARPCs) were able to decrease Peripheral blood mononuclear cell (PBMC) proliferation. (**A**) TLR2-activated-ARPCs were able to decrease the viable count of Con-A activated PBMCs in an independent set of 3 experiments with 3 ARPC clones and PBMC from 3 different healthy donors after both 5 days and 15 days of co-culture. Data from 15 days of co-culture were normalized to 5 days of co-culture in order to compare differences in viable counts. (**B**) BrdU assays showed that LTA-stimulated ARPCs decreased PBMCs’ proliferation rate both after the 5th and 15th day of co-culture. The histograms represent the mean ± SEM. (**C**) PBMCs’ morphology was examined under a light-inverted microscope. Con-A activated PBMCs’ morphology was observed after 5 and 15 days of culture in different conditions. Con-A activated PBMCs formed rounded cellular aggregate after 5 and 15 days of culture in response to the Con-A mitogen effect. When Con-A activated PBMC were co-cultured with ARPCs or LTA-activated ARPCs, a lower number of cellular aggregates was observed both after 5 and 15 days. PBMC images were acquired by an upright microscope with a 10× objective. (**D**) Comparative analysis of apoptosis in Con-A activated PBMCs revealed no significant increase in early and late apoptosis or necrosis when cells were co-cultured with ARPCs and LTA-activated ARPCs after 5 days of co-culture. After 15 days of co-culture, early and late apoptosis increased slightly. Results are representative of 3 independent experiments on 3 different cell lines.

To study the immunomodulatory capacity of ARPCs, we set up a co-culture system in which ARPCs were cultured together with PBMCs but separated from a porous set. PBMCs were activated by concanavalin-A (Con-A) and ARPCs were activated with lipoteichoic acid (LTA), a TLR2 agonist, as previously showed. Cells were co-cultured for 5 or 15 days. We found that renal progenitors were able to significantly decrease the proliferation of activated PBMC both at 5 days of co-culture and at 15 days of co-culture (Figure 1A), those this only applied when they were stimulated by the LTA.

After 5 days of co-culture with activated ARPCs, PBMCs’ proliferation rate significantly decreased (Figure 1A,B) This was also made evident by the morphological observation of Con-A activated PBMCs (Figure 1C). We also studied whether the decreased proliferation could be due to an apoptotic process. We found that no apoptotic cells were present in Con-A activated PBMC cultures after 5 days. In co-culture with ARPCs and activated ARPCs, Co-A activated PBMCs showed no significant increase of apoptotic cells. After 15 days of co-culture with ARPCs and activated ARPCs, PBMCs showed a slight but still not significant increase of early apoptotic cells. 

### 2.2. ARPCs Have Immunomodulatory Effects on Treg and Double Negative T Cells

**Figure 2:**
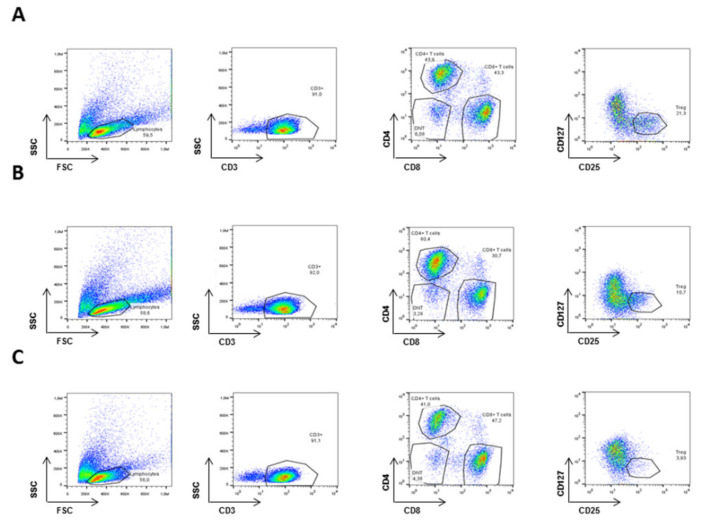
Gating strategy for T cell analysis. PBMC isolated from the peripheral blood of healthy donors, were activated for 24 h with Concanavalin and were cultured alone (**A**) or co-cultured with ARPCs (**B**) or with LTA-activated ARPCs (**C**) for 5 or 15 days and analyzed by flow cytometry. The figure is representative of one experiment showing the gating strategy used to identify lymphocytes (left panel), CD3^+^ T cells and T cells subsets: CD4^+^ T cells, CD8^+^ T cells, DN T cells and Tregs.

We then studied whether the ARPCs immunomodulatory effect was the same for all the T cell subpopulations (Figure 2). T cells subsets (Total T cells, CD4^+^ T cells, CD8^+^ cells, Tregs, and DN T cells) were analyzed by flow cytometry and the gating strategy is shown in Figure 2. 

**Figure 3:**
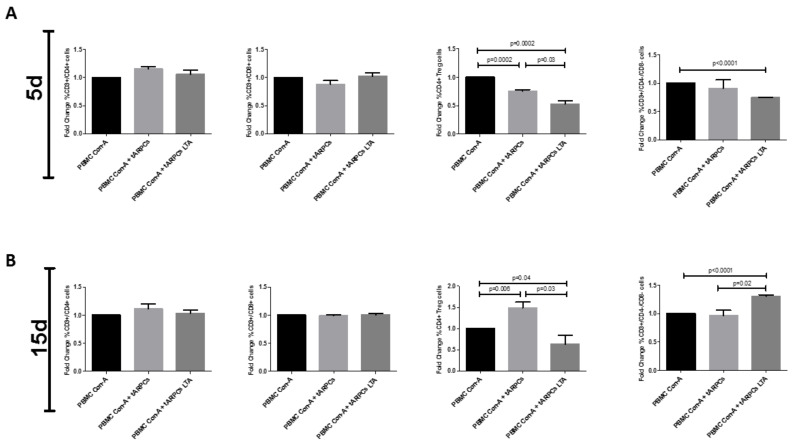
ARPCs are able to modulate Tregs and DN T cells. After 5 and 15 days of co-culture with ARPC or LTA-activated ARPCs, no changes were observed in both CD4^+^ T cells and CD8^+^ T cell subsets. After 5 days of co-culture, LTA acti-vated-ARPCs were able to significantly decrease the % of Tregs and DN T cells in Con-A activated PBMC (**A**). After 15 days of co-culture, LTA activated-ARPCs significantly decreased the % of Tregs and increased the % of DN T cells in Con-A activated PBMC (**B**). Data represent the fold change of the PBMC percentage respect to PBMC Con-A (basal). Data are representative of four independent experiments (means ± SEM).

We found that the renal progenitors did not influence the cell proliferation neither of the CD4^+^ nor of the CD8^+^ subset and neither after 5 days nor after 15 days of co-culture, even when we stimulated the ARPCs with LTA (Figure 3A, B). However, ARPCs had a strong immunomodulation effect on Treg subsets, both at 5 days (Figure 3C) and at 15 days (Figure 3D) of co-culture and particularly when activated by LTA. Moreover, we found that activated ARPCs induced a significant decrease of DN T cells (CD3^+^ CD4^−^ CD8^−^ T cells; Figure 3C) at 5 days of co-culture and instead an increase of the same cells after 15 days of co-culture (Figure 3D). The DN T cell modulation was induced only when ARPCs were activated and not without LTA stimulation. Numerical data and Fold Changes are summarized in Supplementary Tables S1 and S2.

**Figure 4:**
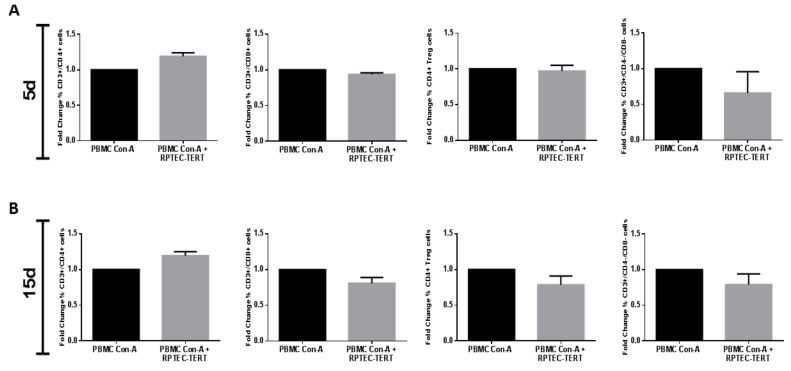
The immunomodulation effect was specific for ARPCs. No significant changes in the % of T cell subsets were observed when ConA activated PBMC were co-cultured with RPTECs for 5 (**A**) or 15 days (**B**). Data represent the fold change of the PBMC percentage with respect to PBMC Con-A. Data are representative of three independent experiments (means ± SEM).

The immunomodulatory effect was specific of the ARPCs since, in the same experimental conditions, the RPTEC in place of ARPCs did not produce any effect (Figure 4 and Supplementary Tables S3 and S4). 

### 2.3. ARPC Communicate with T Cell by Means of Specific Chemokines

**Figure 5:**
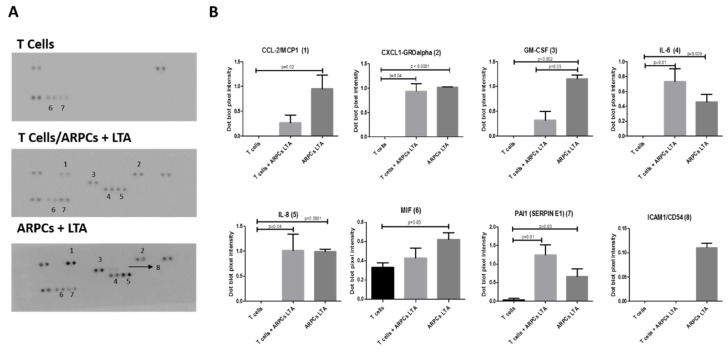
Activated ARPCs secreted a specific pattern of cytokines. The Human Cytokine Array detects 36 human cytokines in cell culture supernatants. The arrows and numbers indicate the cytokine array dot blots. (**A**) Dot blot pixel intensity is graphed. (**B**) Data are representative of three independent experiments (means ± SEM).

To study the mechanisms by which ARPCs induced the immunomodulatory effect, we performed a proteome profile, taking advantage of our in vitro model of co-culture. In particular, we studied the parallel expression of 36 selected human cytokines and chemokines in the supernatants of each of the co-culture conditions: CD3+ T cells alone, CD3+ T cells in co-culture with ARPCs, and CD3+ T cells in co-culture with ARPCs previously stimulated with LTA. The list of screened proteins is reported in Supplementary Table S1. We focused principally on chemokines that were expressed in activated ARPCs and/or in co-culture conditions but that were not expressed, or little expressed in supernatants of T cells alone. We found that Plasminogen Activator Inhibitor 1(PAI1; Serpin E1), C-X-C Motif Chemokine Ligand 1(CXCL1; GRO-α), Colony Stimulating Factor 2 (CSF2; GM-CSF), C-C Motif Chemokine Ligand 2 (CCL2; MCP1), Interleukin 6 (IL-6), Interleukin 8 (IL-8), Macrophage Migration Inhibitory Factor (MIF), and Intercellular Adhesion Molecule 1 (ICAM1) proteins were expressed in these conditions (Figure 5). 

PAI1 and IL-6 were not highly expressed in CD3 T cells alone, but they increased in supernatants of activated ARPCs and increased to a greater extent in co-culture supernatants. CXCL1 and IL-8 were expressed at the same level in both activated ARPCs and in the co-culture but they were not expressed in the T cells alone. GM-CSF and MCP1 were expressed in activated ARPCs and, to a lesser extent, in co-culture conditions. ICAM 1 was expressed only in activated renal progenitors but not in co-culture nor in T cells alone. Finally, MIF was expressed in all the three conditions but at higher levels in the presence of renal progenitors. 

### 2.4. Chemokines Validation

**Figure 6:**
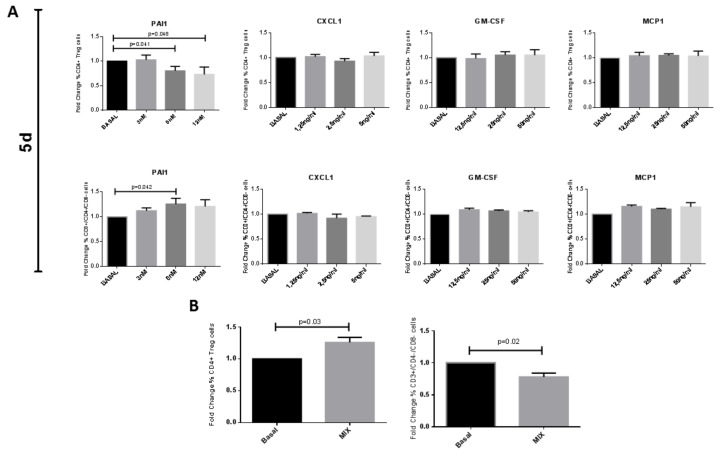
A cocktail of cytokines mediated the immunomodulatory effect of ARPCs after 5 days. (**A**) Con-A activated PBMC were exposed to different crescent concentrations of PAI1, CXCL1, GM-CSF, and MCP1 for 5 days. Percentages of Tregs and DN T cells were analyzed using flow cytometry. PAI1 was able to significantly decrease the % of Tregs at 6 nM and 12 nM but significantly increased DN T cells at 6 nM. None of CXCL1, GM-CSF, and MCP1 were able to significantly modulate T cell subsets alone. (**B**) When Con-A activated PBMC were exposed to the 4 cytokines in mix for 5 days, an immunomodulatory effect was observed on Tregs and DN subsets, similar to the ARPCs co-culture effect. Data represent the fold change of PBMC percentage respect to PBMC Con-A (Basal). Data are representative of six independent experiments (means ± SEM).

We then further validated secreted proteins and studied whether PAI1, CXCL1, GM-CSF, or MCP1, alone or in combination, were able to modulate the Tregs and the DN T cells. We exogenously stimulated PBMCs with these proteins for 5 days. We used three different chemokines concentration: 3 nM, 6 nM, and 12 nM for PAI1; 1.25 ng/mL, 2.5 ng/mL, and 5 ng/mL for CXCL1; 12.5 ng/mL, 25 ng/mL, and 50 ng/mL for GM-CSF and MCP1. PAI1 decreased Tregs both at 6 nM and at 12 nM but increased DN T cells at 6 nM (Figure 6A). The CXCL1, GM-CSF, and MCP1 proteins (at 5 ng/mL and 50 ng/mL, respectively) did not give any significant modulation either on Treg cells or on DN T cells (Figure 6). We then tried to stimulate activated PBMCs by means of a cocktail of the four chemokines (6nM PAI1, CXCL1 5 ng/mL, GM-CSF 50 ng/mL, MCP1 50 ng/mL). Following the 5 days of stimulation, the Treg cells tended to increase whereas the DN T cells significantly decreased, as in co-culture experiments (Figure 6B and Supplementary Table S5).

**Figure 7:**
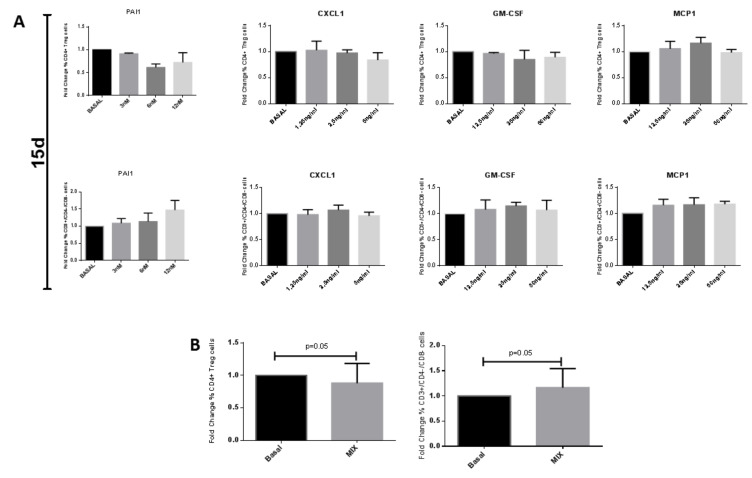
A cocktail of cytokines mediated the immunomodulatory effect of ARPCs after 15 days. (**A**) Con-A activated PBMC were exposed to different crescent concentration of PAI1, CXCL1, GM-CSF and MCP1 for 15 days. None of PAI1, CXCL1, GM-CSF, and MCP1 alone were able to significantly modulate T cell subsets. (**B**) When Con-A activated PBMC were exposed to the 4 cytokines in a mix for 15 days, an immunomodulatory effect was observed on Treg and DN subsets, perfectly comparable to the ARPCs’ co-culture effect. Data represent the fold change of PBMC percentage respect to PBMC Con-A (Basal). Data are representative of six independent experiments (means ± SEM).

In addition, we stimulated PBMCs with the single chemokines at the same concentrations or with their mix for 15 days. PAI1, CXCL1, and GM-CSF tended to decrease, whereas MCP1 tended to increase Tregs. However, all chemokines tended to increase DN T cells. Anyway, none of the chemokines induced a significant modulation of Tregs or DN T cells (Figure 7A). On the contrary, the stimulation with the chemokine mix for 15 days induced a decrease of Tregs and an increase of DN T cells as T cells in co-culture for 15 days with activated ARPCs (Figure 7B and Supplementary Table S6). 

## 3. Discussion

Some types of stem cells, as MSCs, can also repair the tissues by modulating inflammation and regulating the immune response in reaction to an injury [11,22].

In this study, we show for the first time that ARPCs also have an immunomodulatory capacity and in particular that they can modulate Tregs and DN T cells proliferation. We also show that renal progenitors need to be activated first to actuate these functions. As previously demonstrated [5,16], ARPCs can be triggered by TLR2 agonists such as LTA that are a major constituent of the cell wall of gram-positive bacteria and are important for stimulating innate immune responses to gram-positive bacteria [23]. This is something similar to what happens with the MSC, whose immunosuppressive capacity is not constitutive but regulated by inflammatory microenvironment: the quantities and types of inflammatory chemokines differ considerably throughout the beginning and progression of inflammatory diseases and therefore critically affect the triggering of immunoregulation by MSCs, thus controlling ultimately the immunoregulatory effects of these cells [13,14].

We found that activated ARPCs can inhibit the PBMC proliferation in general, but when we analyzed PBMC subpopulations, only Tregs and DN T cells were significantly modulated (Figure 3). If ARPCs perceived the inflammation by means of the LTA binding on TLR2 [1,5], then the Tregs generation was inhibited both in the short term (5 days) and the long term (15 days). 

Considering that both the Tregs and the DN T cells compose 1–5% of all T-cells in healthy mice and human subjects [24,25], the shifts in the percentage of Tregs and DN Ts are significant. The LTA-stimulated ARPCs induced a mean Treg decrease from 5.39 cell % of the basal condition to 2.92% in the co-culture at 5 days and from 6.8 to 4.5 cell % at 15 days (Fold changes of 0.55 and 0.67, respectively). Moreover, TLR2 activated ARPCs induced a mean DN T cells decrease from 7.97 cell % of the basal condition to 5.94% in the co-culture at 5 days and an increase from 6.25 to 9.13 DN T cell % at 15 days (Fold changes of 0.74 and 1.24, respectively). 

**Figure 8:**
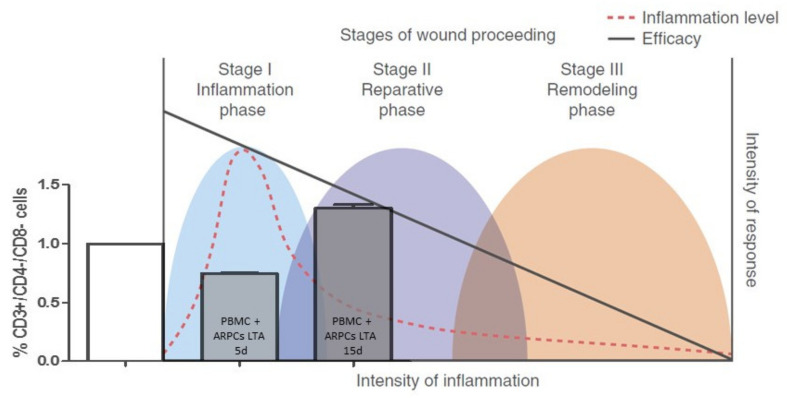
ARPCs can mediate immunomodulation and affect inflammatory state. “The physiological response to tissue damage can be divided into three phases: inflammatory, reparative, and remodeling. During this process, inflammatory status (defined as the types and concentrations of cytokines and cells of the immune system present) changes considerably: proinflammatory influences (red dashed line) are dominant in the inflammatory, infection-fighting phase and diminish in the reparative and remodeling phases that follow, which allows wound healing. In the context of the intensity of the immune response (right vertical axis), the inflammatory response (red dashed line) fluctuates during the wound-healing process. Such changes in inflammation substantially alter the effects of mesenchymal stem cells (MSC)-meditated immunomodulation, which results in a variable correlation between the intensity of inflammation and efficacy of MSC treatment (solid black line)”. Such changes in inflammation are also affected by ARPC-meditated immunomodulation. ARPCs cause DN T cell decrease at the initial phase, promoting inflammation, and DN T cell increase in the late inflammation stages, favoring the inflammation quenching. Adapted by permission from Springer Nature: Springer Nature, NATURE IMMUNOLOGY, Plasticity of mesenchymal stem cells in immunomodulation: pathological and therapeutic implications, Ying Wang, Xiaodong Chen, Wei Cao, and Yufang Shi, COPYRIGHT 2014.

Instead, if ARPCs were not activated by LTA, they can inhibit Tregs anyway, even if to a lesser extent, in the short term; in contrast, they increased Tregs generation in the long term. This type of trend is typical of the physiological response to tissue damage. It can be divided into three stages: inflammatory, reparative, and remodeling. Throughout this process, inflammatory status (expressed as the kinds and quantity of cytokines and cells of the immune system present) varies considerably: the intensity of inflammation is high in the infection-fighting stage and decreases in the subsequent reparative and remodeling stages that allow wound healing [15]. Accordingly, ARPCs cause Tregs and DN T cells to decrease at the initial phase, promoting inflammation and DN T cell increases in the late inflammation stages, favoring the inflammation quenching (Figure 8). However, if the trigger of inflammation persists, ARPCs cause a further decrease of Tregs, contributing to developing a detrimental inflammatory state. The capacity to regulate the Tregs is not new for stem cells: MSC can modulate these kinds of T cells too [26,27]. Instead, to our knowledge, what is new is the capacity of stem/progenitor cells to modulate the recently discovered population of DN T cells. These are highly potent suppressor cells both in mice and in humans. DN T cells can act as regulatory T cells and are able to avoid allograft rejection, graft-versus-host disease, and auto- immune diabetes [28]. They have a homeostatic role in suppressing undue immune responses that are deleterious to the host [29,30]. Moreover, recent studies indicate that DN T cells are also potent regulators of B cells, DCs, and NK cells [28]. DN Tregs are also antigen-specific suppressor cells. Specifically, they use trogocytosis to regulate T cells with the same antigen specificity and this is a unique feature that makes them appealing for a possible cellular therapy, for transplantation, and for autoimmunity [24,28,31]. Recently, the massive presence of this unconventional subset of T cells in normal and ischemic kidneys has been observed both in humans and in mice [32]. Although their role remains partially unknown, in mice models of acute kidney injury (AKI), these cells act as early responders of injury. They significantly expanded within 3 h from injury and decreased after 72 h from injury [33]. Proliferative DN T cells expressed potent anti-inflammatory cytokines in order to modulate tissue immune response [33]. Our data showed that ARPCs activated with LTA decreased the DN T cells at 5 days, favoring the initial inflammation in some way but then in the long term (15 days) renal progenitors increased the DN T cell formation (Figure 3), plausibly to locally avoid or limit prolonged immune response and autoimmunity. 

We also tried to understand what soluble factors, secreted by ARPCs, were involved in the T cell modulation. By using a proteome array of 36 chemokines, we found PAI1, CXCL1/GRO-α, GM-CSF, MCP1, IL-6, IL-8, and MIF that were expressed in the co-culture condition and not by the T cells alone (Figure 5). MCP1, IL-6 and IL-8 had been found modulated by LTA in ARPCs in our previous study [1] and here we also confirmed their upregulation in co-culture with T cells. The IL-8 and MCP1 act as T-lymphocyte chemoattractant [34,35,36,37] and IL-6 is involved in T cell proliferation and differentiation [38,39]. 

Interestingly, GMCSF has an immunomodulatory effect on Tregs regulating their homeostasis and expansion [40,41]. It was completely absent in T cells alone but secreted in large quantity by ARPCs. MIF is an immunomodulatory protein capable to stimulate the TNF-α release and inducing leukocyte recruitment [42,43]. It was expressed at high levels in activated ARPCs. Moreover, several studies showed that in murine models, CXCL1 have a crucial role in the response to infections by migrating neutrophils [44] and to inflammatory response in AKI [45]. Recent studies associated CXCL1 with the balance of Tregs in lungs [46,47]. Finally, PAI1, which is also called Serpin E1, functions as a chemotactic factor of mast cells and upregulating intercellular adhesion molecule type 1 (ICAM1) expression during inflammation [48]. In a mouse model of sepsis, PAI1 acts directly on CD25 levels, modulating the Tregs recruitment in lungs [49]. Remarkably, MIF and PAI1 are expressed by DN T cells, together with RANTES, promoting a T-cell cytokine-enhancing effects that is involved in processes like autoimmune disease and aging, mediated by CD4+ and CD8+ T cells [50]. We showed that PAI1 and MIF are expressed by T cells alone, even if at low levels compared to ARPCs alone. In co-cultures of T cells and activated ARPCs, PAI1 and MIF levels significantly increased. 

In addition, we show that stimulating PBMC with the combination of 4 chemokines, PAI1, CXCL1, MCP1, and GMCSF, had an immunomodulatory effect on Tregs and DN T cells. This effect is very similar to the effect that we observed in co-culture both at 5 and 15 days, suggesting that ARPCs secreted these chemokines as a specific pattern for immunomodulation (Figure 6, Figure 7). As one of the limits of proteome array is the lack of information about the concentration of chemokine in the supernatants of cell cultures, we used a concentration of chemokines similar to those measured in human sera during the inflammatory process, and this could explain the increased levels of Tregs observed after 5 days of stimulation with the mix of chemokines.

## 4. Materials and Methods 

### 4.1. Co-Culture Experiments

Human ARPCs were isolated from portions of normal-appearing cortex of patients who underwent radical or partial nephrectomy for renal clear-cell carcinoma, and were characterized as previously described [1,5,51]. The study was conducted in accordance with the Declaration of Helsinki, and the protocol was approved by the Independent Ethics Committee of the Azienda Ospedaliero Universitaria Policlinico Consorziale of Bari (Italy), Protocol number: 0049338/07/06/2019 issued on 7 June 2019. Informed consent was obtained from all participants included in the study. hTERT immortalized human renal proximal tubular epithelial cell line, was purchased from EverCyte (EverCyte GmbH, Vienna, Austria). Peripheral blood mononuclear cells (PBMCs) that were isolated by gradient centrifugation with the Ficoll-Hypaque method from buffy coats of healthy donors, were selected from our research repository. ARPCs, RPTECs, CD3^+^ T cells, and PBMCs were maintained in their recommended media: endothelial cell growth medium (Lonza, Basel, Switzerland) supplemented with 20% fetal bovine serum (FBS), Prox-Up (EverCyte GmbH) without FBS, and RPMI1640 (

Merck Life Science S.r.l., Milano, Italy) supplemented with 10% FBS, 100 U/mL penicillin, and 100 U/mL streptomycin, 4 mM L-glutamine, 0.1 mM non-essential amino-acids, 10 mM HEPES, 1 mM sodium pyruvate, and 50 U/mL rhIL-2, respectively. 

All PBMC and ARPCs or RPTECs co-cultures were performed in PBMC media. Before co-culture, PBMC were activated through incubation with 5 ng/mL Concanavalin A (Con-A, Sigma Aldrich) for 24 h and ARPCs were activated by triggering TLR2 for 24 h with 30 ug/mL of Lipoteichoic acid (LTA, Sigma Aldrich). 

For in vitro experiments, PBMC were plated at a density of at 1 * 10^6^ cells/cm^2^, and were incubated in medium alone or in the presence of: 5 ng/mL Concanavalin A (Con-A, Sigma Aldrich) for 24 h; 3 nM, 6 nM, or 12 nM of PAI1 (Sigma Aldrich); 1.25 ng/mL, 2.5 ng/mL or 5 ng/mL of CXCL1 (Novus); 12.5 ng/mL, 25 ng/mL or 50 ng/mL of GMCSF (Millipore); or 12.5 ng/mL, 25 ng/mL, or 50 ng/mL of MCP1 (Sigma Aldrich) for 5 or 15 days. 

For co-culture experiments, ARPCs were seeded on top of 0.4-mm-thick polycarbonate inserts (Costar Corning, Life Sciences, Acton, MA, USA) at 8000 cells/cm^2^ in PBMC medium, and were incubated in medium alone or activated by triggering TLR2 for 24 h with 30 µg/mL of Lipoteichoic acid (LTA, Sigma Aldrich). After 24 h of incubation alone, PBMC and ARPCs were used for co-cultures in PBMC medium for 5 days or 15 days and viable cells were counted by trypan blue dye exclusion (Sigma-Aldrich S.r.l., Milan, Italy) on a Burker chamber. 

### 4.2. Proliferation Assay

Cell proliferation was measured by bromodeoxyuridine (BrdU) incorporation during the last 6 h of 5 and 15 days of culture by a colorimetric immunoassay, according to the manufacturer’s guidelines (Roche Diagnostics, Mannheim, Germany). Untreated cells were used as controls. BrdU incorporated into the DNA was detected using an anti-BrdU peroxidase-conjugated antibody and visualized with a soluble chromogenic substrate. Values were acquired as absorbance at 450 nm—absorbance also occurred at 690 nm. 

### 4.3. Flow Cytometry Analysis

Apoptosis of PBMCs in different conditions after 5 and 15 of co-culture was measured using Annexin-V and 7AAD staining (Beckman coulter, Milan, Italy). PBMC were stained with the following monoclonal antibodies (mAbs) for flow cytometry analysis (FACS): PEVio615-conjugated anti-CD3 (clone REA613), PeVio770-conjugated anti-CD4 (clone REA623), FITC-conjugated anti-CD8 (clone REA734), PE-conjugated anti-CD25 (clone 3G10), and APC-conjugated anti-CD127 (clone REA614) (or their corresponding isotype controls). PBMC were incubated for 20 min with the antibody mixes in the dark at room temperature, washed twice, and resuspended in FACS buffer. All mAbs and their respective isotypes were purchased from Miltenyi Biotec (Bologna, Italy). The anti-CD3 antibody was used to identify total T cells (CD3^+^ cells), then the anti-CD8, anti-CD4, anti-CD-25 and anti-CD127 were used to distinguish T cells subsets: CD4 T cells (CD3^+^ CD4^+^ CD8^−^ T cells), CD8 T cells (CD3^+^ CD4^−^ CD8^+^ T cells) CD4 Treg cells (CD3+ CD4^+^ CD25^+^ CD127^low/−^ T cells) [52], and Double Negative (DN) T cells (CD3^+^ CD4^−^ CD8^−^ T cells). Stained cells were then acquired on a Navios cytometer (Beckman Coulter SRL, Cassina de´Pecchi, Milan, Italy) and analyzed using the Flowjo software (BD Biosciences, San Jose, California). 

### 4.4. Proteome Array

Levels of 36 cytokines from cell supernatant of PBMC, LTA activated-ARPCs and 24 h PBMC- LTA activated-ARPCs co-culture were detected using the Proteome Profiler Human Cytokine Array kit (Cat. No.: ARY005B—R & D System, Minneapolis, MN, USA). The assay was conducted following the manufacturer’s instructions. Dot Blots were quantified using Image J 1.34 Software. 

### 4.5. Statistical Analysis

Statistical analysis was performed using the Student’s t test or ANOVA, as appropriate. A value of *p* > 0.05 was considered significant. Data are expressed as means ± SEM.

## 5. Conclusions 

In summary, we demonstrated for the first time that ARPCs have immunomodulatory properties in response to an inflammatory environment, leading to regulation of Tregs and DN T cells, which are involved in the balance between immune tolerance and autoimmunity. Considering that many renal diseases are characterized by inflammatory infiltrating T cells, which are mostly DN T cells, further investigations would be useful to more extensively study the contribution of ARPCs in modulating immune system during acute and chronic kidney injury.

## Data Availability

The data presented in this study are available in article and supplementary material.

## Supplementary Materials

The following are available online at https://www.mdpi.com/1422-0067/22/1/274/s1, Table S1: Numerical data and Fold Changes for Figure 3A, Table S2: Numerical data and Fold Changes for Figure 3B, Table S3: Numerical data and Fold Changes for Figure 4A, Table S4: Numerical data and Fold Changes for Figure 4B, Table S5: Fold Changes for Figure 6, Table S6: Fold Changes for Figure 7.
