# Supplementary material for: Adult Renal Stem/Progenitor Cells Can Modulate T Regulatory Cells and Double Negative T Cells
Source: Int J Mol Sci. 2020 Dec 29;22(1):274. doi: 10.3390/ijms22010274 (PMC7795073; doi:10.3390/ijms22010274)
Supplement: Supplementary file 1 [file ijms-22-00274-s001.pdf]

Supplementary Table 1: Numerical data and Fold Changes for Figure 3A

	PBMC Con-A	PBMC Con-A +ARPCs	PBMC Con-A + ARPCs LTA	PBMC Con-A	PBMC Con-A +ARPCs (FC)	PBMC Con-A + ARPCs LTA (FC)
**$CD3^+/CD4^+$ cells**	36.5 ± 12.0	42.7 ± 15.1	37.3 ± 10.7	1	1.16 ± 0.03	1.06 ± 0.07
**$CD3^+/CD8^+$ cells**	49.8 ± 13.2	45.6 ± 13.8	49.8 ± 11.6	1	0.88 ± 0.06	1.02 ± 0.06
**$CD4^+$ TREGs cells**	5.4 ± 1.1	4.1 ± 0.9	2.9 ± 0.5	1	0.75 ± 0.03*	0.55 ± 0.04*;**
**$CD3^+/CD4^-/CD8^-$ cells**	7.9 ± 3.1	7.0 ± 3.3	5.9 ± 2.3	1	0.90 ± 0.15	0.74 ± 0.003***

Data are expressed as mean % ± SEM of four independent experiments;
*: p=0.0002 versus PBMC CoA; ** :p=0.003 versus PBMC CoA+ARPCs; ***: p<0.0001 versus PBMC CoA

Supplementary Table 2: Numerical data and Fold Changes for Figure 3B

	PBMC Con-A	PBMC Con-A +ARPCs	PBMC Con-A + ARPCs LTA	PBMC Con-A	PBMC Con-A +ARPCs (FC)	PBMC Con-A + ARPCs LTA (FC)
**$CD3^+/CD4^+$ cells**	40.3 ±7.9	46.8 ±13.22	42.2 ±9.57	1	1.13 ±0.12	1.03 ±0.05
**$CD3^+/CD8^+$ cells**	45.4 ±10.7	44.9 ±10.3	45.22 ±9.79	1	0.98 ± 0.01	1.00 ±0.02
**$CD4^+$ TREGs cells**	6.8 ±1.5	10.4 ±2.2	4.5 ±0.9	1	1.54 ±0.12*	0.67 ±0.13**;***
**$CD3^+/CD4^-/CD8^-$ cells**	6.3 ±2.9	5.7 ±2.5	9.1 ±3.2	1	0.96 ±0.09	1.24 ±0.06$;$$

Data are expressed as mean % ± SEM of four independent experiments
*: p=0.006 versus PBMC CoA; **: p=0.04 versus PBMC CoA; ***: p=0.03 versus PBMC CoA+ARPCs
$: p<0.001 versus PBMC CoA; $$: p=0.02 versus PBMC CoA+ ARPCs

Supplementary Table 3: Numerical data and Fold Changes for Figure 4A

	PBMC Con-A	PBMC Con-A + RPTEC	PBMC Con-A	PBMC Con-A+ RPTEC (FC)
**$CD3^+/CD4^+$ cells**	41.6 ± 16.5	48.6 ± 17.5	1	1.19 ± 0.05
**$CD3^+/CD8^+$ cells**	41.3 ± 17.4	38.1 ± 15.2	1	0.93 ± 0.02
**$CD4^+$ TREGs cells**	6.1 ± 3.6	6.2 ± 4.0	1	0.97 ± 0.08
**$CD3^+/CD4^-/CD8^-$ cells**	11.3 ± 0.7	7.2 ± 2.9	1	0.66 ± 0.30

Data are expressed as mean % ± SEM of three independent experiments

Supplementary Table 4: Numerical data and Fold Changes for Figure 4B

	PBMC Con-A	PBMC Con-A + RPTEC	PBMC Con-A	PBMC Con-A+ RPTEC (FC)
**$CD3^+/CD4^+$ cells**	41.9 ± 15.9	54.7 ± 17.7	1	1.32 ± 0.07
**$CD3^+/CD8^+$ cells**	43.6 ± 17.8	36.6 ± 17.8	1	0.81 ± 0.08
**$CD4^+$ TREGs cells**	6.1 ± 2.3	5.1 ± 2.6	1	0.78 ± 0.12
**$CD3^+/CD4^-/CD8^-$ cells**	8.4 ± 0.06	6.5 ± 0.7	1	0.79 ± 0.15

Data are expressed as mean % ± SEM of three independent experiments

Supplementary Table 5: Fold Changes for Figure 6

	PBMC Con-A	**PBMC Con-A + PAI1 (FC)**			PBMC Con-A + CXCL1 (FC)			**PBMC Con-A + GM-CSF (FC)**			PBMC Con-A + MCP-1 (FC)			**PBMC Con-A + MIX (FC)**
		**3nM**	**6nM**	**12nM**	1.25ng/ml	2.5ng/ml	5ng/ml	**12.5ng/ml**	**25ng/ml**	**50ng/ml**	12.5ng/ml	25ng/ml	50ng/ml	
**$CD4^+$ TREGs cells**	1	1.02 ± 0.10	0.81 ± 0.09*	0.73 ± 0.15*	1.04 ± 0.04	1.02 ± 0.06	1.16 ± 0.06	0.99 ± 0.09	1.09 ±0.06	1.25 ± 0.05	1.07 ± 0.06	1.08 ±0.04	1.20 ±0.09	1.18 ± 0.11**
**$CD3^+/CD4^-/CD8^-$ cells**	1	1.12 ± 0.06	1.26 ± 0.12*	1.26 ± 0.16	1.08 ± 0.05	1.05 ± 0.03	1.11 ± 0.09	1.13 ± 0.05	1.07 ± 0.02	1.15 ± 0.10	1.28 ±0.13	1.10 ±0.01	1.25 ± 0.12	0.88 ± 0.11***

Data are expressed as mean % ± SEM of six independent experiments

*: p=0.04 versus PBMC CoA **: p=0.03 versus PBMC CoA; ***: p=0.02 versus PBMC CoA

Supplementary Table 6: Fold Changes for Figure 7

	PBMC Con-A	**PBMC Con-A + PAI1 (FC)**			PBMC Con-A + CXCL1 (FC)			**PBMC Con-A + GM-CSF (FC)**			PBMC Con-A + MCP-1 (FC)			**PBMC Con-A + MIX (FC)**
**$CD4^+$ TREGs cells**	1	0.99 ± 0.08	0.76 ± 0.09	0.84 ± 0.12	1.25 ± 0.08	0.97 ± 0.04	1.01 ± 0.05	0.96 ± 0.01	0.99 ± 0.16	0.98 ± 0.09	1.12 ± 0.10	1.21 ± 0.09	1.27 ± 0.14	0.74 ± 0.27*
**$CD3^+/CD4^-/CD8^-$ cells**	1	0.99 ± 0.12	1.03 ± 0.19	1.12 ± 0.17	0.91 ± 0.08	0.92 ± 0.14	0.95 ± 0.06	0.95 ± 0.15	1.00 ± 0.13	0.96 ± 0.15	1.07 ± 0.1	0.99 ± 0.17	1.00 ± 0.15	1.24 ± 0.15*

Data are expressed as mean % ± SEM of six independent experiments

*: p≤0.05 versus PBMC CoA
